# Proteomic Analysis Implicates Dominant Alterations of RNA Metabolism and the Proteasome Pathway in the Cellular Response to Carbon-Ion Irradiation

**DOI:** 10.1371/journal.pone.0163896

**Published:** 2016-10-06

**Authors:** Yu Wang, Hua Guan, Da-Fei Xie, Yi Xie, Xiao-Dan Liu, Qi Wang, Li Sui, Man Song, Hong Zhang, Jianhua Zhou, Ping-Kun Zhou

**Affiliations:** 1 Department of Radiation Toxicology and Oncology, Beijing Key Laboratory for Radiation Biology, Beijing Institute of Radiation Medicine, Beijing, China; 2 Department of Heavy Ion Radiation Medicine, Institute of Modern Physics, Chinese Academy of Sciences, Lanzhou 730000, China; 3 iBioinfo Groups, Lexington, Massachusetts 02421, United States of America; 4 Collaborative Innovation Center of Radiation Medicine of Jiangsu Higher Education Institutions, School of Radiation Medicine and Protection, Soochow University, Suzhou, China; 5 China Institute of Atomic Energy, Beijing 102413, China; 6 Department of Neuroregeneration, Nantong University, Nantong, China; National Taiwan University, TAIWAN

## Abstract

Radiotherapy with heavy ions is considered advantageous compared to irradiation with photons due to the characteristics of the Braggs peak and the high linear energy transfer (LET) value. To understand the mechanisms of cellular responses to different LET values and dosages of heavy ion radiation, we analyzed the proteomic profiles of mouse embryo fibroblast MEF cells exposed to two doses from different LET values of heavy ion ^12^C. Total proteins were extracted from these cells and examined by Q Exactive with Liquid Chromatography (LC)—Electrospray Ionization (ESI) Tandem MS (MS/MS). Using bioinformatics approaches, differentially expressed proteins with 1.5 or 2.0-fold changes between different dosages of exposure were compared. With the higher the dosage and/or LET of ion irradiation, the worse response the cells were in terms of protein expression. For instance, compared to the control (0 Gy), 771 (20.2%) proteins in cells irradiated at 0.2 Gy of carbon-ion radiation with 12.6 keV/μm, 313 proteins (8.2%) in cells irradiated at 2 Gy of carbon-ion radiation with 12.6 keV/μm, and 243 proteins (6.4%) in cells irradiated at 2 Gy of carbon-ion radiation with 31.5 keV/μm exhibited changes of 1.5-fold or greater. Gene ontology (GO) analysis, Kyoto Encyclopedia of Genes and Genomes (KEGG) analysis, Munich Information Center for Protein Sequences (MIPS) analysis, and BioCarta analysis all indicated that RNA metabolic processes (RNA splicing, destabilization and deadenylation) and proteasome pathways may play key roles in the cellular response to heavy-ion irradiation. Proteasome pathways ranked highest among all biological processes associated with heavy carbon-ion irradiation. In addition, network analysis revealed that cellular pathways involving proteins such as Col1a1 and Fn1 continued to respond to high dosages of heavy-ion irradiation, suggesting that these pathways still protect cells against damage. However, pathways such as those involving Ikbkg1 responded better at lower dosages than at higher dosages, implying that cell damage would occur when the networks involving these proteins stop responding. Our investigation provides valuable proteomic information for elucidating the mechanism of biological effects induced by carbon ions in general.

## Introduction

Radiotherapy using heavy ions beams or protons is becoming an important component of malignant tumor therapy [1, 2]. Heavy-ion radiation has a number of advantages for cancer radiotherapy over photon therapy. The major advantage is the inverted dose profile, which features a sharp longitudinal dose drop, referred to as the Bragg peak, at the end of the particle range [3]. The increased therapeutic ratio permits dose escalation within the tumor, consequently resulting in improved tumor control. Another advantage is the high linear energy transfer (LET) characteristics of heavy-ion beams [4]. The biological consequences of radiation exposure depend not only on the radiation dose and dose rate but also on the radiation quality. High-LET radiation, such as carbon-ion beam, deposits higher energy in tissues and causes greater damage than low-LET γ- or X-ray irradiation [4, 5]. The radiation energy deposition increases as the LET value increases with increasing transversal depth [6]. The LET value is unique for each heavy ion. The increased biological efficacy of high LET is usually described as the quantity of relative biological effectiveness (RBE) compared to low-LET γ- or X-ray irradiation, which is dependent on the LET value [7, 8]. In the irradiated pre-osteoblast cell line OCT-1, the RBE calculated using survival curves *D*_0_ increased with LET up to 150 keV/μm from 1 for X-rays (0.3–3 keV/μm) to 3.5 for ^56^Fe ions (150 keV/μm), then decreased to 0.67 for ^58^Ni ions with an LET of 905 keV/μm [9]. However, the RBE is a complex quantity that depends not only on the radiation properties but also on biological factors, such as endpoints. Cell cycle regulation of G_2_/M arrest, an immediate response to ionizing radiation-induced DNA damage, appears to be more sensitive to high-LET radiation than cell survival. Significant, LET-dependent G_2_/M arrest was observed in cells exposed to heavy ions. The ratio of G_2_/M-arrested cells after high-LET irradiation at doses resulting in 1% cellular survival were 47, 81, and 78% for carbon-, neon-, and iron-ion radiation, respectively [9]. Data from a study in which the spinal cords of rats were irradiated with single doses of carbon ions suggested a linear relationship between RBE and LET at high doses for late effects [10]. However, the minimum latency time was dose-dependent but not significantly LET-dependent [10]. A differential metabolic response has also been observed for high-LET heavy ions and low-LET γ irradiation. For example, ^56^Fe radiation preferentially altered dipeptide metabolism and induced the upregulation of ‘prostanoid biosynthesis’ and ‘eicosanoid signaling’, interlinked events associated with cellular inflammation, compared to equitoxic exposure to γ radiation [11].

In clinical tumor therapy using heavy ion beams, the skin at the surface of the body is simultaneously irradiated with an entrance plateau irradiation whose LET is lower than at the peak. Therefore, in addition to killing cancer cells, heavy-ion radiotherapy may induce normal tissue damage in terms of both acute and later effects. An abrupt increase/decrease in normal tissue damage in the course of carbon-ion radiotherapy has been reported as a result of changing the number of fractions [12]. Localized deposition of high-LET heavy ions results in a complex network of cellular and molecular responses. Although radiobiological studies have provided some valuable information about the cellular response to high-LET irradiation, e.g., clustered complex DNA damage, a higher RBE value, and lower oxygen effect, the molecular mechanisms underlying the development of such cellular changes and tissue reactions and the large variation in biological effects induced by different values of LET radiation are not fully understood.

Proteomics is a comprehensive technology that permits the identification of a large quantity of proteins with differential expression levels or post-translation modification states. Proteomic studies are important for uncovering the complex biological pathways and molecular networks related to physiological or pathological status or environmental stresses, including ionizing radiation. Interest in proteomic investigations of radiation biology is increasing [13] following the broad application of genomic technology and bioinformatics to studies of the effects of radiation, including dose and dose-rate effects [14, 15], differential end points of cancer and normal tissue [16–20], individual radiation sensitivity, and biomarker identification [20, 21] for radiation biodosimeters or risk assessment. A comprehensive combination of comparative proteomics and advanced bioinformatics would provide valuable insights into the complex biological networks that are influenced by radiation exposure [13, 22–24]. A number of proteomic studies of heavy-ion effects have also been performed recently [25–27]. In the present study, a combination of proteomics and bioinformatics was applied to identify and compare the biological pathways and molecular networks involved in the response of mouse skin fibroblast cells to carbon-ion irradiation with LET values equivalent to the entrance plateau and Bragg peak. We identified protein and RNA metabolism (proteasome pathways, RNA splicing, RNA degradation) as potentially playing key roles in regulating cellular responses to heavy ion irradiation. In addition, network analysis revealed that cellular pathways involving proteins such as Col1a1 and Fn1 continue to respond to high dosages of heavy-ion irradiation, suggesting that these pathways continue to protect cells against damage. By contrast, pathways such as ikbkg1 exhibit improved responses at lower dosages than higher dosages, implying that cell damage occurs when these proteins stop responding. The results of this study may facilitate the design of improved strategies to treat tumors while protecting normal tissues by combining low dosages of heavy-ion irradiation with suppression of the expression of proteins such as ikbkg1.

## Materials and Methods

### Cells culture and chemicals

Wild type mouse embryo fibroblast (MEF) cells were obtained from Prof. Tom Hei’s Laboratory in Columbia University, New York. The MEF cells were isolated from 13.5-d embryos of wild type C57BL/6J mouse strain and the Ethics Statement was previously described [28]. MEF cells were frozen down and preserved in liquid nitrogen. MEF Cells were removed from liquid nitrogen and thawed quickly in a 37°C water bath, and were maintained and grown in DMEM (HyClone) supplemented with 15% heat-inactivated fetal bovine serum (HyClone), 100 units/ml penicillin and 100 μg/ml streptomycin, and cultured in a humidified incubator at 37°C with 5% CO_2_. Passage 3 cultures of the thawed cells from liquid nitrogen freezing were used for experiments, and the maximum passage of cells used in this study was no more than 8 after thawed from liquid nitrogen freezing. All chemicals were purchased from Sigma (St. Louis, MO) unless otherwise noted.

### Irradiation procedure

Carbon-ion irradiation was performed at room temperature at the Heavy Ion Accelerator Center (HIRFL) of the Institute of Modern Physics, Lanzhou, Chinese Academy of Sciences (Lanzhou, China), with 300Mev/u monoenergetic carbon ion beams. The penetration depth was adjusted by changing the thickness of the absorber. The cells were irradiated with carbon-ion beams with the LET value 12.6KeV/μm at the entrance plateau. The penetration depths of the ions in water were 15.3 cm (the plateau region of the Bragg curve), and LET value was 31.5 KeV/μm. The cells were irradiated by 0.2 Gy or 2 Gy of carbon-ion beams with the LET value of 12.6 keV/μm or irradiated by 2 Gy of carbon-ion beams with the LET value of 31.5 keV/μm. Three irradiated cells samples were performed for each group.

For X-ray irradiation was performed using the RS2000 X ray biological irradiator (RadSource, Suwanee, GA, USA) at the dose rate of 118 cGy/min. The machine settings: 160 kV 25 mA, 0.3 mm of copper filter, and 45 keV of mean energy.

### Protein preparation, digestion and iTRAQ labeling

MEF cells collected 4 h after carbon-ion irradiation or the sham control were washed twice in ice-cold PBS. The cell pellets were homogenized in iTRAQ dissolution buffer to isolate total protein for iTRAQ labeling according to the manufacturer’s instructions. The protein samples were prepared, separately, from the cells irradiated by 0 Gy (sham irradiation control), 0.2 Gy or 2 Gy of carbon-ion beams with an LET of 12.6 keV/μm (LET12.6–0.2Gy or LET12.6-2Gy), and 2 Gy of carbon-ion beams with an LET of 31.5 keV/μm (LET31.5-2Gy). Protein digestion was performed using trypsin (Promega) at 37°C overnight, and the resulting peptide mixtures of each sample were separately labeled at room temperature for 1 h using the reagents of the iTRAQ Multi-Plex Kit (Applied Biosystems, Foster City, CA) according to the manufacturer’s instructions. For labeling, each iTRAQ reagent was dissolved in 50 μl of ethanol and added to the respective peptide mixture. After labeling, the samples were mixed and dried using a rotary vacuum concentrator.

### Peptide fractionation

The iTRAQ-labeled peptides were fractionated by SCX chromatography using an AKTA Purifier system (GE Healthcare). The dried peptide mixture was reconstituted and acidified with 2 ml of Buffer A (10 mM KH_2_PO_4_ in 25% ACN) and loaded onto a PolySULFOETHYL 4.6 x 100 mm column (5 μm, 200 Å, PolyLC Inc.). The peptides were eluted at a flow rate of 0.7 ml/min with a gradient of 5%–8% Buffer B (98% ACN-2% H2O) for 1 min, 8–32% Buffer B for 24 min, 32%–95% Buffer B for 2 min, 95% buffer B for 4 min, and 95%–5% Buffer B for 1 min. The elutions were monitored by absorbance at 214 nm, and fractions were collected every 1 min. The collected fractions were then desalted. Each fraction was concentrated by vacuum centrifugation and reconstituted in 1.9%ACN/98%H_2_O/0.1% (v/v) trifluoroacetic acid. All samples were stored at -80°C until LC-MS/MS analysis.

### Liquid chromatography (LC)—electrospray ionization (ESI) tandem MS (MS/MS) analysis by Q Exactive

Experiments were performed on a Q Exactive mass spectrometer (Thermo Fisher Scientific) coupled to an EASY-nLC-1000 (Thermo Fisher Scientific). A 10-μl aliquot of each fraction was injected for nanoLC-MS/MS analysis. The peptide mixture (5 μg) was loaded onto a C18 reversed-phase column (Thermo Scientific Easy Column, 10 cm long, 75 μm inner diameter, 3 μm resin) in Buffer A (0.1% formic acid) and separated using a linear gradient of Buffer B (80% acetonitrile and 0.1% formic acid) at a flow rate of 250 nl/min controlled by IntelliFlow technology over 140 min. MS data were acquired using a data-dependent top10 method, which dynamically selected the most abundant precursor ions from the survey scan (300–1800 m/z) for higher-energy collisional dissociation (HCD) fragmentation. Determination of the target value was based on predictive Automatic Gain Control (pAGC).

### Sequence database searching and data analysis

MS/MS spectra were searched using the MASCOT engine (Matrix Science, London, UK; version 2.2) embedded into Proteome Discoverer 1.3 (Thermo Electron, San Jose, CA) against the Uniprot Human database and the decoy database. For protein identification, the following options were used: Peptide mass tolerance = 20 ppm; MS/MS tolerance = 0.1 Da; Enzyme = Trypsin; Missed cleavages = 2; Fixed modification: Carbamidomethyl (C); iTRAQ 8plex (K), iTRAQ 8plex (N-term); Variable modification: Oxidation (M); FDR ≤ 0.01.

### Bioinformatic analysis

#### Data analysis

The proteomics data were searched against the built-in Rattus database using Thermo Scientific Proteome Discoverer (v1.2). The expression profile data are the means of three experiments data for each detected protein of each irradiation group. Proteins that changed more than 1.5-fold are reported.

#### Gene set analysis

Gene sets were downloaded from Molecular Signatures Database v3.1 (http://www.broadinstitute.org/gsea/msigdb/index.jsp) and Panther Classification System v8.1 (http://www.pantherdb.org/). *P* values were calculated by selecting genes with changes of greater than 1.5-fold and applying a hypergeometric distribution. The *P* value was further modified by multiplying the exponential by the ratio of the gene sets.

#### Network analysis

The network analysis was generated from Exploratory Gene Association Networks (EGAN, http://akt.ucsf.edu/EGAN/) by selecting genes with changes of greater than 1.5-fold.

#### Cell survival

The MEF cells were washed with 0.02% EDTA and treated with 0.02% trypsin for 6 min. The trypsin was then neutralized with the growth medium and the cells were collected by centrifugation and resuspended in growth medium. The cell concentrations were determined using a haemocytometer, and an appropriate number of cells (3 × 10^2^–2 × 10^4^) were plated onto 60 mm diameter plastic petri dishes. When the cells were adhered onto the dishes post-approximate 4 h culture, cells irradiations were performed using Carbon-ion radiation of HIRFL, Lanzhou, or X ray irradiator as described above. Six dishes were plated for each radiation dose. After incubation for 14 days, the cells were fixed and stained using gentian violet (1% solution containing 5% formaldehyde) and the number of colonies containing over 50 cells was counted. Four replicate experiments were performed for X-ray irradiation. Two experiments were performed for carbon-ion irradiation, but six dishes were prepared for each radiation dose at each of two cell densities.

$$\text{The plating efficiency}\;=\;\frac{\text{colony number of non-irradiated control cells}}{\text{plated cell number of non-irradiated control cells}}$$

$$\text{The survival fraction}\;=\;\frac{\text{colony number of a dose irradiated cells}}{\text{plated cell number of a dose irradiated cells × plating efficiency }}$$

### Immunoblotting hybridization

The cells were harvested at given times after carbon-ion irradiation or the sham control and washed twice with ice-cold PBS. The cell pellets were treated with lysis buffer, and total protein was isolated. The protein (50 μg) was resolved by SDS-PAGE (8%) and then transferred onto a polyvinylidene fluoride (PVDF) membrane for immunoblotting hybridization analysis. Antibodies against RanGAP (ab4784), SOX14 (ab4947), PI-3-kinase class 3(ab73262) and Histone 1 H2A1H were purchased from ABcam^®^. γH2AX (Ser 139) antibody (JBW301, #05–636) was purchased from Millipore, and β-actin antibody (C4, sc-47778) was purchased from Santa Cruz Biotechnology.

## Results

### Proteomics analysis

Total protein was extracted from MEF cells 4 h after exposure to heavy carbon-ion beams. The proteins were separated by 2-D electrophoresis and analyzed by mass spectrometry. The proteomics data were searched against the built-in Rattus database using Thermo Scientific Proteome Discoverer (v1.2). Cellular sensitivity to carbon-ion radiation was determined and compared with the X-ray by the assay of colony forming ability. The dose—response curves for cell killing (S1A Fig) and the survival parameter *D*_37_ (S1B Fig) showed that the carbon-ion radiation killed more cells as compared with X-ray. Notably, the relative biological effect (RBE), calculated with *D*_37_, of carbon-ion with the LET of 31.5 keV/μm is higher than that of carbon-ion with the LET of 12.6 keV/μm (S1B Fig).

**Fig 1:**
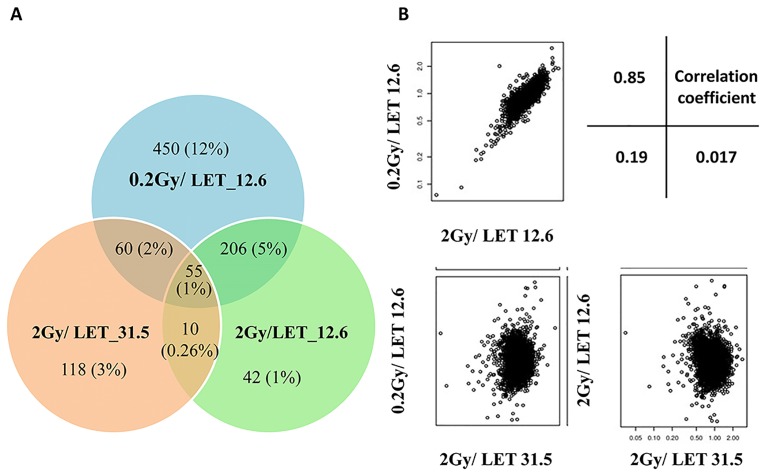
Proteins differentially expressed in cells exposed to 0.2 Gy low and 2 Gy high doses of carbon ions with different LET values. (A) Venn diagram showing distinct and overlapping proteins differentially regulated among these three groups of carbon ion-irradiated cells. (B) Correlation analysis of protein expression changes among the three groups. The corresponding correlation coefficients are shown in the top right corner.

A total of 3818 proteins have been identified in carbon-ion irradiated cells. Compared to the control (0 Gy), 771 (20.2%) proteins in 0.2 Gy-irradiated cells and 313 proteins (8.2%) in 2 Gy-irradiated cells with carbon-ion radiation with the LET of 12.6 keV/μm, and 243 proteins (6.4%) in cells irradiated at 2 Gy by carbon-ion radiation with a relative higher LET of 31.5 keV/μm, exhibited a change of 1.5-fold or greater (Fig 1A). Further analysis indicated that many more proteins exhibited decreased expression than increased expression in all cells exposed to heavy-ion irradiation. This distribution pattern was more prominent in cells exposed to 0.2 Gy of 12.6 keV/μm carbon ion beams, with 603 proteins exhibiting 1.5-fold decreased expression and 168 proteins exhibiting 1.5-fold increased expression. The upregulated proteins in each irradiation group and their overlaps with other groups are presented in S1–S3 Tables.

As shown in S1 Table, 28.6% (48/168) of the proteins upregulated by 0.2 Gy of carbon-ion radiation with an LET of 12.6 keV/μm were also upregulated by 2 Gy of carbon-ion radiation with the same LET value. However, only four of these proteins, transport and Golgi organization 6 (Tango6), COBW domain-containing 1 (Cbwd1), atlastin GTPase 3(Atl3), and transforming growth factor beta regulated gene 4 (Tbrg4), were upregulated by 2 Gy of the relative higher-LET carbon-ion radiation (31.5 keV/μm). Among the 70 proteins upregulated by 2 Gy of carbon-ion radiation with an LET of 12.6 keV/μm, 47 (67.1%) were also upregulated by 0.2 Gy of carbon-ion radiation with the same LET value (S2 Table), and only 9 (12.9%) were upregulated by 2 Gy of 31.5 keV/μm carbon-ion radiation. The proteins upregulated by 2 Gy of both 12.6 and 31.5 keV/μm of carbon-ion beams included Tango6, SRY-box-containing gene 14 (Sox14), glycerol-3-phosphate acyltransferase, Dync1li2, UDP glucuronosyltransferase 1 family polypeptide A9 (Ugt1a9), Cbwd1, alpha-1-B glycoprotein (A1bg), breakpoint cluster region, and phosphoinositide-3-kinase class 3. However, among the 136 proteins upregulated by 2 Gy of carbon-ion radiation with an LET of 31.5 keV/μm, only 8 (5.9%) were regulated by 2 Gy of 12.6 keV/μm carbon-ion irradiation (S3 Table), including the three proteins describe above that were also upregulated by 0.2 Gy of 12.6 keV/μm carbon-ion irradiation, Tango6, Cbwd1 and Atl3.

**Fig 2:**
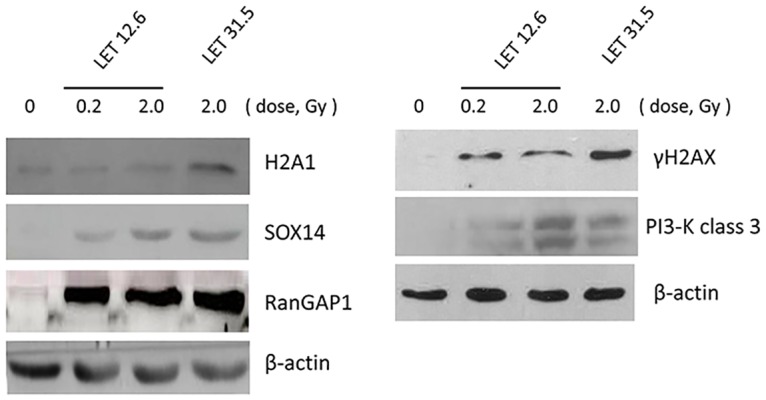
Analyses of proteins expression changes by immunoblotting hybridization. The altered expression levels of H2A1, SOX14, RanGAP1, γH2AX, and PI3K-III were verified in the cells after irradiation with carbon ions at an LET value of 12.6 keV/μm or 31.5 keV/μm. The cell extracts for immunoblotting were prepared at 4 h after irradiation. β-actin expression was used as a sample loading control.

Correlation analysis revealed a good relationship between the protein expression changes at the 0.2 Gy low and 2 Gy high doses of carbon-ion radiation with an LET of 12.6 keV/μm but not between ion irradiation at different LET values (Fig 1B). To validate the results of the proteomic analysis, we selected five proteins (H2A1, SOX14, RainGAP1, γH2AX, and PI3K-III) for confirmation by Western blotting analysis. Consistent with the proteomic profiling results, the expression of H2A1, SOX14, RanGAP1, H2AX, and PI3K-III in MEL cells significantly increased after exposure of the cells to carbon-ion irradiation in a dosage- or LET-dependent manner (Fig 2).

### Gene set analysis of differentially expressed proteins

The effects of heavy ions on cells are dosage dependent. To investigate how irradiation regulates a functionally interconnected and interdependent set of biological processes, including proteasome pathways, cell cycle, apoptosis, DNA damage response, and, more recently, mRNA splicing, processing and stability [29, 30], we conducted gene set analysis using several different methodologies, including gene ontology (GO) analysis, Kyoto Encyclopedia of Genes and Genomes (KEGG) analysis, Munich Information Center for Protein Sequences (MIPS) analysis, and BioCarta analysis.

Tables 1–6 detail the categories that were significantly enriched in differentially expressed proteins corresponding to heavy carbon-ion irradiation, with proteasome pathways always ranked #1 (p-value = 2.21E-09), DNA damage response ranked #5, mRNA processing and splicing ranked #4 and #6 and cell division ranked #8 (p-value = 3.01E-6). Glycolysis/gluconeogenesis pathways ranked #2 (BioCarta analysis, Table 3) and #7 (KEGG analysis, Table 2). Our gene set analysis revealed highly significant overlap among our top-ranked groups and previously published results. For example, 13 of the 16 proteins we identified as involved in the 20S proteasome exhibited a change in expression of 1.5-fold or greater in cells irradiated at 0.2 Gy. It is not surprising that proteasome pathways ranked highest of all biological processes associated with heavy carbon-ion irradiation in all gene set analyses (GO, KEGG, MIPS and BioCarta), with significant adjusted p-values. Although not previously closely associated with carbon-ion irradiation in any systematic manner, 38 of 127 proteins from the RNA splicing pathway (KEGG analysis) were identified as significantly regulated, corresponding to 30% of the genes in the pathway.

**Table 1:** Top-ranked pathways by GO analysis.

Rank	Pathway	Pathway size	Observed	Ratio	*P*-value
1	CYTOPLASM	2130	165	0.077	4.6E-64
2	RNA_BINDING	259	43	0.166	5.5E-30
3	RNA_SPLICING	91	22	0.242	1.1E-19
4	RNA_PROCESSING	173	30	0.173	7.0 E-22
5	RIBONUCLEOPROTEIN_COMPLEX	143	26	0.182	1.1E-19
6	MRNA_PROCESSING_GO_0006397	73	16	0.219	5.4E-14
7	RNA_SPLICING_FACTOR_ACTIVITYTRANSESTERIFICATION_MECHANISM	19	7	0.368	1.4E-08
8	MRNA_METABOLIC_PROCESS	84	17	0.202	3.6E-14
9	NUCLEUS	1428	104	0.073	2.6E-37
10	DRUG_BINDING	16	6	0.375	1.4E-07
11	CYTOPLASMIC_PART	1383	100	0.072	1.3E-35
12	PROTEIN_FOLDING	58	13	0.224	9.3E-12

**Table 2:** Top-ranked pathways by KEGG analysis.

Rank	Pathway	Pathway size	Observed	Ratio	*P*-value
1	KEGG_SPLICEOSOME	127	38	0.299	1.4E-10
2	KEGG_PROTEASOME	47	16	0.340	6.4E-06
3	KEGG_RNA_DEGRADATION	59	18	0.305	1.2E-05
4	KEGG_GLUTATHIONE_METABOLISM	50	13	0.26	0.0015
5	KEGG_FRUCTOSE_AND_MANNOSE_METABOLISM	34	9	0.264	0.0088
6	KEGG_FOCAL_ADHESION	200	27	0.135	0.012
7	KEGG_GLYCOLYSIS_GLUCONEOGENESIS	62	11	0.177	0.048
8	KEGG_PROTEIN_EXPORT	24	6	0.25	0.050
9	KEGG_PENTOSE_PHOSPHATE_PATHWAY	27	6	0.222	0.082
10	KEGG_PYRUVATE_METABOLISM	40	7	0.175	0.14
11	KEGG_DRUG_METABOLISM_OTHER_ENZYMES	51	8	0.157	0.16
12	KEGG_ASCORBATE_AND_ALDARATE_METABOLISM	25	5	0.2	0.16

**Table 3:** Top-ranked pathways by BioCarta analysis.

Rank	Pathway	Pathway size	Observed	Ratio	*P*-value
1	BIOCARTA_PROTEASOME_PATHWAY	28	16	0.571	1.7E-20
2	BIOCARTA_GLYCOLYSIS_PATHWAY	10	5	0.5	7.7E-07
3	BIOCARTA_P53HYPOXIA_PATHWAY	23	7	0.304	2.4E-07
4	BIOCARTA_PTDINS_PATHWAY	23	7	0.304	2.4E-07
5	BIOCARTA_NDKDYNAMIN_PATHWAY	19	5	0.263	2.9E-05
6	BIOCARTA_INTEGRIN_PATHWAY	38	7	0.184	9.0E-06
7	BIOCARTA_FREE_PATHWAY	10	3	0.3	0.0008
8	BIOCARTA_GABA_PATHWAY	10	3	0.3	0.0008
9	BIOCARTA_GLEEVEC_PATHWAY	23	5	0.217	7.7E-05
10	BIOCARTA_CK1_PATHWAY	17	4	0.235	0.0003
11	BIOCARTA_P35ALZHEIMERS_PATHWAY	11	3	0.272	0.0011
12	BIOCARTA_SET_PATHWAY	11	3	0.272	0.0011

**Table 4:** Top-ranked pathways by MIPS analysis.

Rank	Pathway	Pathway size	Observed	Ratio	*P*-value
1	MIPS_PA28_20S_PROTEASOME	16	13	0.812	2.3E-17
2	MIPS_26S_PROTEASOME	22	15	0.681	1.8E-15
3	MIPS_PA700_20S_PA28_COMPLEX	36	16	0.444	4.1E-09
4	MIPS_SPLICEOSOME	142	35	0.246	3.7E-08
5	MIPS_ESCRT_III_COMPLEX	10	6	0.6	9.0E-06
6	MIPS_TNF_ALPHA_NF_KAPPA_B_SIGNALING_COMPLEX_10	10	6	0.6	9.0E-06
7	MIPS_C_COMPLEX_SPLICEOSOME	80	20	0.25	3.9E-05
8	MIPS_EXOSOME	11	5	0.454	0.0013
9	MIPS_CDC5L_COMPLEX	30	8	0.266	0.0090
10	MIPS_HCF_1_COMPLEX	19	4	0.315	0.0098
11	MIPS_LARGE_DROSHA_COMPLEX	20	3	0.3	0.0011
12	MIPS_12S_U11_SNRNP	15	3	0.333	0.0011

**Table 5:** Top-ranked pathways by PID analysis.

Rank	Pathway	Pathway size	Observed	Ratio	*P*-value
1	PID_ILK_PATHWAY	45	9	0.2	0.0335
2	PID_IGF1_PATHWAY	30	6	0.2	0.0955
3	PID_IL1PATHWAY	34	6	0.176	0.1437
4	PID_PI3KPLCTRKPATHWAY	36	6	0.166	0.1694
5	PID_LIS1PATHWAY	28	5	0.178	0.1861
6	PID_AVB3_INTEGRIN_PATHWAY	75	9	0.12	0.2229
7	PID_UPA_UPAR_PATHWAY	42	6	0.142	0.2479
8	PID_NFAT_3PATHWAY	54	7	0.129	0.2552
9	PID_RHOA_PATHWAY	45	6	0.133	0.2866
10	PID_SYNDECAN_1_PATHWAY	46	6	0.130	0.2993
11	PID_LYMPHANGIOGENESIS_PATHWAY	25	4	0.16	0.3098
12	PID_FOXOPATHWAY	49	6	0.122	0.3364

**Table 6:** Top-ranked pathways by REACTOME analysis.

Rank	Pathway	Pathway size	Observed	Ratio	*P*-value
1	REACTOME_REGULATION_OF_MRNA_STABILITY_BY_PROTEINS_THAT_BIND_AU_RICH_ELEMENTS	82	29	0.353	2.1E-11
2	REACTOME_DESTABILIZATION_OF_MRNA_BY_AUF1_HNRNP_D0	51	19	0.372	2.3E-08
3	REACTOME_REGULATION_OF_ORNITHINE_DECARBOXYLASE_ODC	48	18	0.375	4.8E-08
4	REACTOME_MRNA_SPLICING	109	29	0.266	1.1E-07
5	REACTOME_VIF_MEDIATED_DEGRADATION_OF_APOBEC3G	51	18	0.352	2.0E-07
6	REACTOME_CDK_MEDIATED_PHOSPHORYLATION_AND_REMOVAL_OF_CDC6	47	17	0.361	2.7E-07
7	REACTOME_CROSS_PRESENTATION_OF_SOLUBLE_EXOGENOUS_ANTIGENS_ENDOSOMES	47	17	0.361	2.7E-07
8	REACTOME_ACTIVATION_OF_NF_KAPPAB_IN_B_CELLS	63	20	0.317	4.7E-07
9	REACTOME_AUTODEGRADATION_OF_THE_E3_UBIQUITIN_LIGASE_COP1	49	17	0.346	6.7E-07
10	REACTOME_P53_INDEPENDENT_G1_S_DNA_DAMAGE_CHECKPOINT	49	17	0.346	6.7E-07
11	REACTOME_SCFSKP2_MEDIATED_DEGRADATION_OF_P27_P21	54	18	0.333	7.1E-07
12	REACTOME_SCF_BETA_TRCP_MEDIATED_DEGRADATION_OF_EMI1	50	17	0.34	1.0E-06

Although a number of signaling relationships exist among ubiquitin, the cell cycle, and DNA damage, few of these proteins overlapped with either GO categories. However, using literature-based annotation and network analysis, we identified a densely interconnected network among these proteins from the proteasome, the cell cycle, DNA damage and ubiquitin ligase conjugation pathways, RNA splicing, and RNA metabolisms.

### Network analysis of differentially expressed proteins

**Fig 3:**
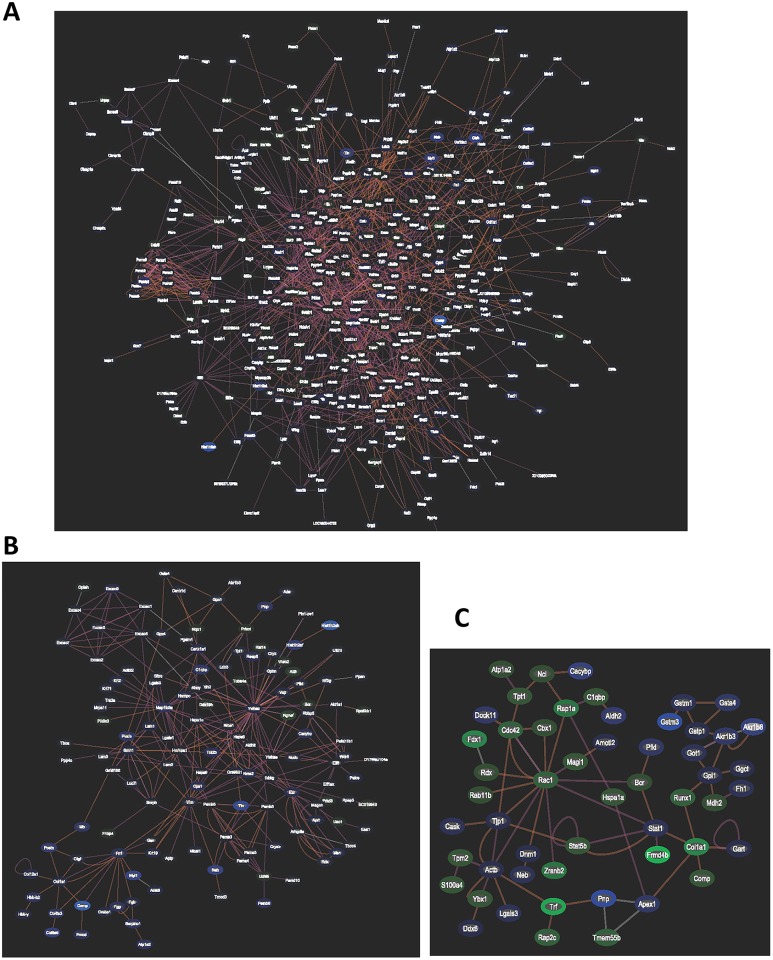
Network analysis of differentially expressed proteins with 1.5-fold changes. (A) The network of differentially expressed proteins from the cells irradiated by 0.2 Gy of carbon-ion radiation with an LET of 12.6 keV/μm. (B) The network of differentially expressed proteins from the cells irradiated by 2 Gy of carbon-ion radiation with an LET of 12.6 keV/μm. (C) The network of differentially expressed proteins from the cells irradiated by 2 Gy of carbon-ion radiation with an LET of 31.5 keV/μm.

To examine how the proteins affected by heavy carbon-ion irradiation, Exploratory Gene Association Networks analysis (EGAN, http://akt.ucsf.edu/EGAN/) of proteins exhibiting a 1.5- to 2.0-fold increase or decrease in expression after irradiation was performed (Fig 3). We observed that the initial response of cells to a lower dosage of irradiation was dramatic, with many genes exhibiting a 1.5- to 2.0-fold increase or decrease in expression. The network of gene expression after a lower dosage of irradiation of 0.2 Gy was complex (Fig 3A). At higher dosages of irradiation, the number of genes exhibiting a 1.5- to 2.0-fold increase or decrease in expression was significantly reduced, and the regulatory network became much simpler (Fig 3B and 3C). We deduced that, under high-LET heavy-ion irradiation, higher dosages of 2 Gy might have exceeded the thresholds of cellular tolerance, leading to irreversible damage.

**Fig 4:**
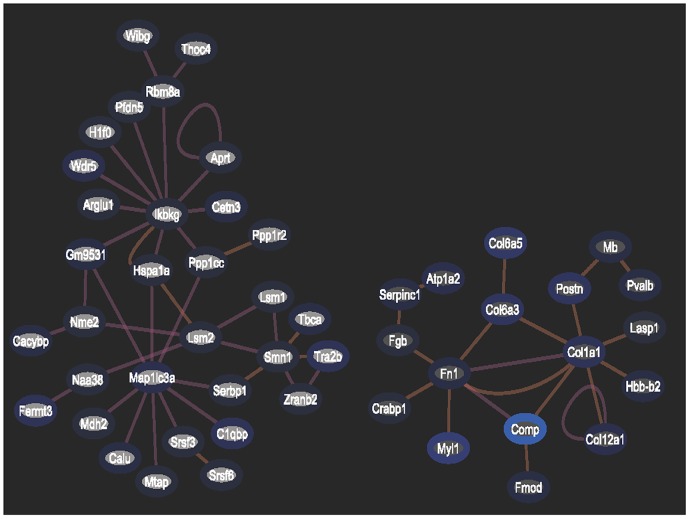
Network analysis of differentially expressed proteins with changes of 2.0-fold or greater in irradiated by 0.2 Gy of carbon-ion radiation with an LET of 12.6 keV/μm.

**Fig 5:**
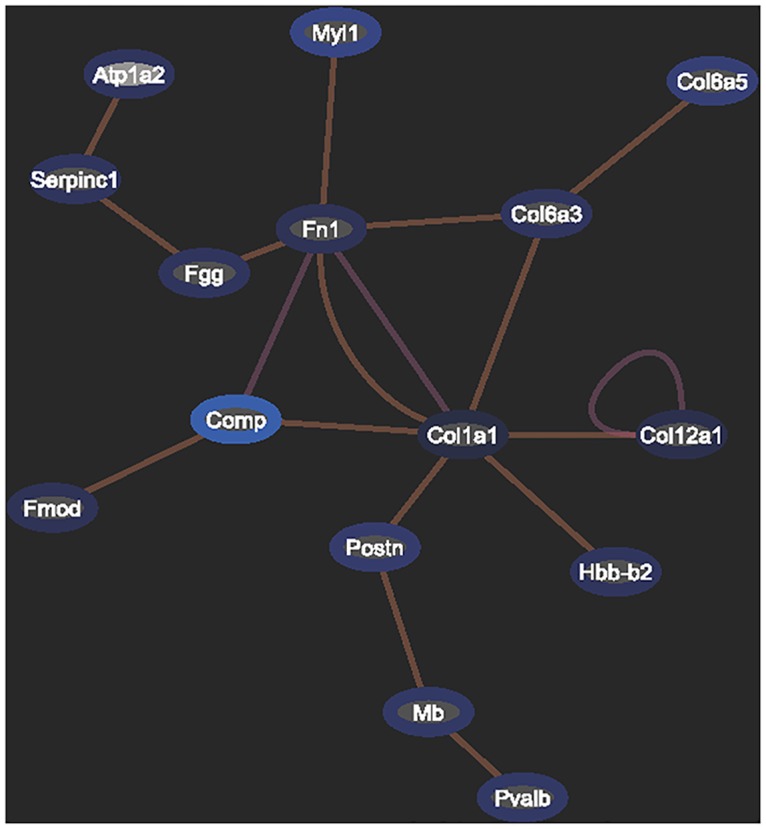
Network analysis of differentially expressed proteins with changes of 2.0-fold or greater in the cells irradiated by 2 Gy of carbon-ion radiation, with an LET of 12.6 keV/μm.

Analysis of genes exhibiting a 1.5 to 2.0-fold increase or decrease in expression demonstrated that a lower dosage (0.2 Gy) triggered the regulation of several gene expression networks, suggesting that these networks of gene regulation may play protective roles in response to irradiation (Fig 4). Some pathways, such as those involving ikbkg1, exhibited superior responses only at the lower dosage and not at higher dosages. However, the increase in irradiation to 2 Gy might have exceeded the thresholds of regulation of these networks, with the exception of those related to Col1a1 (Fig 5). Col1a1 expression level also changed: increased to 2.141 fold of the control in the cells irradiated by 2 Gy of 31.6 kev/μm carbon-ion radiation, while decreased to 0.307 and 0.498 fold in the cells irradiated by 0.2 Gy or 2 Gy of 12.6 kev/μm carbon-ion radiation, respectively (S3 Table).

## Discussion

Double-strand breaks (DSBs) are critical lesions of genomic DNA induced by ionizing radiation. Compared with low LET γ-rays, high-LET proton and heavy-ion radiation induces more complicated clustered DNA damage [31, 32]. In mammalian cells, a DNA DSB in the nuclear genome promptly initiates the phosphorylation of histone H2AX at Ser139 to generate γH2AX foci in megabase regions localized around each individual break. The protein γH2AX is an essential and efficient coordinator of the recognition and repair of DNA damage to ensure the maintenance of genomic stability. The formation of nuclear γH2AX foci is a critical immediate-early event in DNA damage signaling in cells after exposure to ionizing radiation, from low-LET γ-rays [33–35] and X-rays [36, 37] to high-LET radiation, e.g., α-particles [38, 39] and heavy ions [33, 34, 40, 41]. Because γH2AX foci appear early at damage sites in a dose-dependent manner, γH2AX is widely considered a sensitive biomarker for IR-induced DSBs. In this study, the γH2AX level was increased in cells exposed to carbon-ion radiation. After irradiation at the same dose (2 Gy), a greater increase in the level of γH2AX was induced in the cells exposed to the higher-LET carbon-ion radiation of 31.5 keV/μm than in the cells exposed to the radiation with an LET of 12.6 keV/μm. Higher LET radiation produces a higher RBE. Therefore, this result could be considered a form of quality control for the irradiated samples in this proteomic analysis. As mentioned above, various potent biological effects are produced by different qualities of LET radiation; these effects are usually described by the term RBE. The greatest advantage of using a heavy-ion beam used for cancer treatment is its physical properties, which allow sharp dose gradients and relative dose escalation, maximizing the dose to the tumors and minimizing the dose to normal organs [2]. In addition, the radiation energy deposited to normal organs and tissues is usually a relatively lower LET radiation with a lower RBE value, which consequently produces lighter injury compared to the radiation injury to the tumors. In this study, the radiosensitivity assay of colony forming ability demonstrated that the carbon-ion radiation killed more cells as compared with X-ray. Moreover, the RBE calculated with *D*_37_ was dependent with of LET values of carbon ions, which of carbon-ion with the LET of 31.5 keV/μm is higher than that of carbon-ion with the LET of 12.6 keV/μm. The differential biological effects produced by different values of LET radiation could be attributed to the induction of a series of mechanistic networks. Previous reports on lower-LET γ- or X-ray irradiation have demonstrated that IR-inducible expression changes either at the transcriptional level [42–45] or the protein level [14, 22, 46, 47] are dose-dependent or dose-specific. Bing and colleagues compared the biological effects induced by low-LET X-rays and high-LET carbon-ion radiation in HeLa cells by proteomic analysis and observed a significant difference in the alterations to mechanistic pathways related to dysregulated proteins between carbon-ion and X-ray irradiation [26]. The analysis of biological processes by searching the DAVID database further demonstrated that most of the affected biological processes in the 2 Gy carbon group differed from those affected in the 2 Gy X-ray group. However, proteomic analyses comparing heavy-ion irradiation with different LET values are scarce. The total number and distribution of the upregulated and downregulated proteins in this proteomics analysis indicate that the changes in expression patterns were more prominent in cells exposed to 0.2 Gy of irradiation of the carbon ions of 12.6 keV/μm. The analysis of genes with a 1.5- to 2.0-fold increase or decrease in expression demonstrated that a lower dosage (0.2 Gy) triggered the regulation of several gene expression networks, suggesting that these networks of gene regulation may play protective roles in response to irradiation. In this study it was found that a higher dose (2 Gy) and a higher LET (31.5 KeV/μm) samples produced lower protein responses, implying that heavy cell damage would occur when the networks involving these cellular protective proteins could stop responding. However, when the radiation dose was increased to 2 Gy and the survival rate decreased to 17%, the regulatory thresholds of some of these networks may have been exceeded, with the exception of the network related to Col1a1. Some pathways, such as the one involving ikbkg1, which located at the central node of a related interacting network (Fig 4), exhibited greater responses at the lower dosage than at higher dosages.

As shown in S1 Table, 28.6% (48/168) of the proteins upregulated by 0.2 Gy of carbon-ion radiation with an LET of 12.6 keV/μm were also upregulated by 2 Gy of carbon-ion radiation with the same LET value. Clearly, larger overlaps of proteomic profiling responses were induced between cells treated with different doses (0.2 and 2 Gy) of the same quality (LET value) of heavy-ion irradiation than by different qualities of heavy-ion radiation at the same dose level. The profile of downregulated proteins displayed the same tendency of LET dependence. Notably, the radiation quality factor of the heavy-ion radiation reached at the tumor tissues is an important physical parameter affecting the outcomes of heavy-ion therapy.

The bioinformatic analysis indicated that RNA and protein metabolism might play key roles in regulating cellular responses to heavy-ion irradiation. The extensive concentration of proteins involved in RNA splicing, RNA stability, and RNA processing and metabolism (Tables 1, 2 and 6) among the differentially expressed proteins suggests an unexpected theme for the regulatory effects of heavy carbon-ion irradiation. For example, the changed proteins included 3' to 5' exoribonuclease, RNA destabilization involving KSRP, proteins that bind AU-rich elements, BRF1, hnRNP D0(AUF1), hnRNPUL1,and a number of other RNA binding proteins associated with RNA splicing (Rbmxl1, Rbm8a, Rbm33, Cirbp, etc) and mRNA deadenylation pathway. Change of RNA metabolisms such as alternative splicing and destabilization, is an effective means of directly regulating mammalian cells in response to DNA damage stress. Tresini et al. reported that UV-induced DNA damage triggers specific and profound changes in spliceosome organization in which the R-loop-dependent activation of ATM signaling plays a key role [48]. DNA repair is a critical cellular response to radiation injury and is required to promote cell survival and maintain genomic stability. Alternative transcription products of several DNA repair genes, including RRM2B and XPC, have been detected in cells after exposure to ionizing radiation [49]. Interestingly, Sprung et al. observed a large number of genes exhibiting alternative transcription initiation in response to ionizing radiation when an exon array platform was used to characterize IR-induced transcriptional expression products [50]. Recently, some RNA-binding proteins have been reported to play the direct role in regulating DDR, which include the heterogeneous nuclear ribonucleoprotein U-like proteins 1 (hnRNPUL1). In present study, hnRNPUL1 was found downregulated in irradiated cells. As a member of hnRNP family, hnRPUL1 can bind to mRNA and plays role in RNA transport, processing and export. In addition to function in RNA metabolism, hnRPUL1 has also been demonstrated to involve in DDR. Hong et al reported that hnRPUL1 could associated with PARP1 and recruited to DNA double-strand breaks (DSBs) sites in a PARP1-mediated poly (ADP-ribosyl) ation dependent manner. Knockdown of hnRPUL1 enhanced the recruitment of PARP1 to DSBs sites [51]. Polo et al have identified that hnRNPUL1 and -2 acted as binding partners for the DSB sensor complex MRE11-RAD50-NBS1 (MRN) in regulation of DNA-end resection, and hnRNPUL1 and -2 were recruited to DNA damage in an interdependent manner that requires MRN [52]. The interaction with NBS1 and recruitment to the sites of DNA damage was regulated by the arginine methylation of hnRNPUL1 [53].

There have been a number of reports about the effects of modulating proteasome activity on the radiosensitivity of cancer cells [54–57]. In this study, KEGG analysis (Table 2), BioCarta analysis (Table 3) and MIPS analysis (Table 4) all demonstrated that differentially expressed proteins in proteasome pathways always ranked first among altered proteins in the proteomic profiles corresponding to heavy carbon-ion irradiation injury. Our investigations have advanced the knowledge of the protein metabolic response to heavy ions.

Obviously, the DNA damage response (DDR) of intricate molecular signals networks is immediately triggered after irradiation, and a series of cellular responses are evolved to counteract DNA damage and cell injury. The early cellular responses, initiated immediately and persisted for 24h or longer after irradiation, include DNA repair, cell cycle arrest, apoptosis, autophagy, etc [58, 59]. In presented study, the proteomic expression profiles were identified 4h post-irradiation, at which time the DNA repair was still undergoing, and cell cycle arrest, cell death or survival signals could also be initiated. Unexceptionally, all these processes are tightly regulated by a series of DDR signaling proteins. The major molecular events include the activation or inactivation, relocation, protein-protein interaction, altered expression levels and degradation of some critical DDR proteins. RNA metabolism and proteasome pathway are two major ways to regulate the activity and cellular amount of proteins. Here we demonstrated that the alterations of RNA metabolism and proteasome pathway ranked forefront among all the categories significantly enriched in differentially expressed proteins at 4h post carbon-ion irradiation. Taken together of the previous reports mentioned above, the RNA metabolism and proteasome pathway does result in significant biological consequences and play a fundamental role in the cellular response to carbon-ion irradiation. The more precious mechanisms underlying the responses of RNA metabolism to ionizing radiation injury remain unclear. Undoubtedly, the alterations of RNA metabolism, including alternative pre-mRNA splicing and degradation, enhance transcriptome complexity and proteome diversity, which not only contribute to cancer development and progression but also have significant biological consequences affecting the therapeutic response of cancer cells to radiation and chemotherapy.

## Conclusions

A combination of proteomics and bioinformatics was applied to identify and compare the biological pathways and molecular networks altered by carbon-ion irradiation with differential LET values and different dosages. A large differential proteomic response was observed for carbon ions with different LET values. This distribution pattern was more prominent in cells exposed to 0.2 Gy than to the higher dosage of 2 Gy. Protein (proteasome pathways) and RNA (RNA splicing, RNA degradation) metabolism were identified as playing potentially key roles in regulating cellular responses to heavy-ion irradiation. In addition, network analysis revealed that cellular pathways involving proteins such as Col1a1 and Fn1 continue to respond to high dosages of heavy-ion irradiation, suggesting that these pathways continue to protect cells against damage. Our investigation provides valuable advanced proteomic information for elucidating the mechanism of biological effects induced by carbon ions in general.

## Supporting Information

S1 Fig Survival curves of colony-forming ability assay and the RBE for carbon-ion irradiation. (TIF)

S1 Table The upregulated proteins by 0.2 Gy of the 12.6 KeV/μm carbon ion beams and their overlaps with other irradiation groups (black characters on pink background). (PDF)

S2 Table The upregulated proteins by 2 Gy of 12.6 KeV/μm carbon ions and their overlaps with other irradiation groups (black characters on pink background). (PDF)

S3 Table The upregulated proteins by 2 Gy of 31.5 KeV/μm carbon ions and their overlaps with other irradiation groups (black characters on pink background). (PDF)
