# Supplementary figures and images for: Proteomic Analysis Implicates Dominant Alterations of RNA Metabolism and the Proteasome Pathway in the Cellular Response to Carbon-Ion Irradiation
Source: PLoS One. 2016 Oct 6;11(10):e0163896. doi: 10.1371/journal.pone.0163896 (PMC5053480; doi:10.1371/journal.pone.0163896)

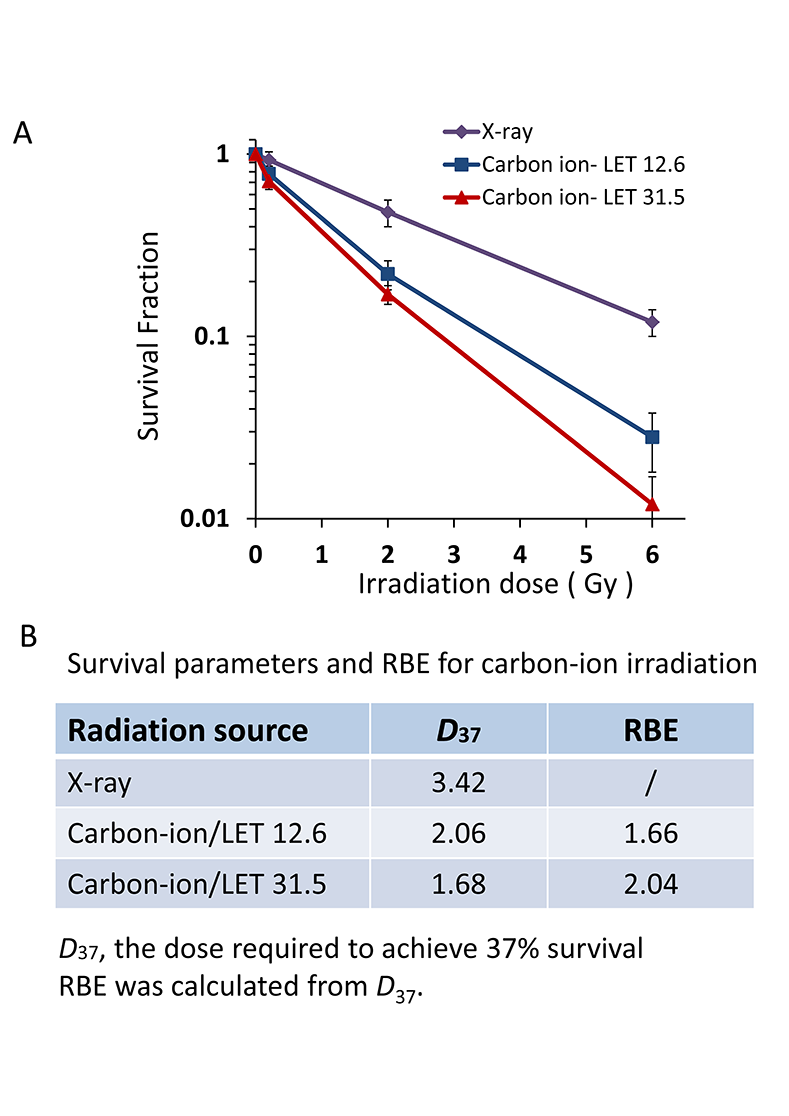

Supplement: S1 Fig — (TIF) [file pone.0163896.s001.tif]
