# Supplementary material for: Proteomic Analysis Implicates Dominant Alterations of RNA Metabolism and the Proteasome Pathway in the Cellular Response to Carbon-Ion Irradiation
Source: PLoS One. 2016 Oct 6;11(10):e0163896. doi: 10.1371/journal.pone.0163896 (PMC5053480; doi:10.1371/journal.pone.0163896)
Supplement: S1 Table — (PDF) [file pone.0163896.s002.pdf]

S1 Table The upregulated proteins by 0.2 Gy of the 12.6 KeV/μm carbon ion beams and their overlaps with other irradiation groups

The upregulated proteins by 0.2 Gy irradiation of carbon ion beams with the LET of 12.6 KeV/μm, and their partial overlaps with other irradiation groups (LET12.6- 2 Gy and LET31.5- 2Gy) (black characters on pink background)

Mouse Gene ID	Symbol	Description	Changed fold		
			LET12.6		LET31.5
			0.2Gy	2Gy	2Gy
216363	Rab3ip	RAB3A interacting protein	2.855	1.894	1.043
69155	1810030O07Rik	RIKEN cDNA 1810030O07 gene	2.567	1.925	1.243
67542	Cog6	component of oligomeric golgi complex 6	2.37	1.81	1.098
75786	Ckap5	cytoskeleton associated protein 5	2.326	2.218	1.332
69150	Snx4	sorting nexin 4	2.299	2.475	0.942
217337	Srp68	signal recognition particle 68	2.295	2.124	1.081
272538	Tango6	transport and golgi organization 6	2.25	3.173	1.818
71799	Ptcd1	pentatricopeptide repeat domain 1	2.048	1.43	0.87
19387	Rangap1	RAN GTPase activating protein 1	1.979	1.172	0.808
109168	Atl3	atlastin GTPase 3	1.954	1.706	0.684
15982	Ifrd1	interferon-related developmental regulator 1	1.951	1.15	1.089
237542	Osbpl8	oxysterol binding protein-like 8	1.915	1.481	1.024
19364	Rad51d	RAD51 homolog D	1.908	1.284	0.825
20419	Shcbp1	Shc SH2-domain binding protein 1	1.904	1.391	0.896
107182	Btaf1	BTAF1 RNA polymerase II, B-TFIID transcription factor-associated, (Mot1 homolog, S. cerevisiae)	1.904	1.379	1.249
59025	Usp14	ubiquitin specific peptidase 14	1.895	1.424	1.012
80748	BC004004	cDNA sequence BC004004	1.888	1.518	1.293
56207	Uchl5	ubiquitin carboxyl-terminal esterase L5	1.884	1.543	0.984
29858	Pmm1	phosphomannomutase 1	1.878	1.257	1.201
234733	Ddx19b	DEAD (Asp-Glu-Ala-Asp) box polypeptide 19b	1.872	1.633	0.798
226043	Cbwd1	COBW domain containing 1	1.866	1.643	1.62
20775	Sqle	squalene epoxidase	1.861	1.423	1.095
16202	Ilk	integrin linked kinase	1.827	1.441	1.031
70549	Tln2	talin 2	1.826	1.72	1.003
69276	Sec62	SEC62 homolog (S. cerevisiae)	1.816	1.596	0.904
15033	H2-T18	histocompatibility 2, T region locus 18	1.805	1.419	1.118
227737	Fam129b	family with sequence similarity 129, member B	1.77	1.695	0.676
16211	Kpnb1	karyopherin (importin) beta 1	1.769	1.393	0.914
22017	Tpmt	thiopurine methyltransferase	1.768	1.434	1.347
107358	Tm9sf3	transmembrane 9 superfamily member 3	1.757	1.402	0.918
12443	Ccnd1	cyclin D1	1.755	1.438	0.715
67673	Tceb2	transcription elongation factor B (SIII), polypeptide 2	1.746	1.548	1.39
69596	Ap5s1	adaptor-related protein 5 complex, sigma 1	1.742	1.402	0.837

		subunit			
63913	Fam129a	family with sequence similarity 129, member A	1.739	1.421	0.94
18753	Prkcd	protein kinase C, delta	1.739	1.565	1.201
72080	Sapcd2	suppressor APC domain containing 2	1.738	1.333	0.865
56041	Uso1	USO1 vesicle docking factor	1.735	1.627	1.05
20466	Sin3a	transcriptional regulator, SIN3A (yeast)	1.728	1.33	0.654
100503235	LOC100503235	ubiquitin-conjugating enzyme E2 E1-like	1.728	1.479	0.809
11938	Atp2a2	ATPase, Ca++ transporting, cardiac muscle, slow twitch 2	1.715	1.418	1.134
229725	Clcc1	chloride channel CLIC-like 1	1.714	1.388	0.868
60525	Acss2	acyl-CoA synthetase short-chain family member 2	1.713	1.444	0.781
73699	Ppp2r1b	protein phosphatase 2 (formerly 2A), regulatory subunit A (PR 65), beta isoform	1.713	1.464	1.041
57342	Parva	parvin, alpha	1.713	1.519	1.145
20166	Rtkn	rhotekin	1.71	1.502	0.939
235406	Snx33	sorting nexin 33	1.706	1.383	1.104
66092	Ghitm	growth hormone inducible transmembrane protein	1.701	1.823	1.132
68274	4930547C10Rik	RIKEN cDNA 4930547C10 gene	1.694	0.988	0.846
217995	Heatr1	HEAT repeat containing 1	1.694	1.89	1.07
67130	Ndufa6	NADH dehydrogenase (ubiquinone) 1 alpha subcomplex, 6 (B14)	1.693	1.5	1.25
433956	Heatr2	HEAT repeat containing 2	1.691	1.807	1.187
69019	Spcs1	signal peptidase complex subunit 1 homolog (S. cerevisiae)	1.686	1.447	1.257
70699	Nup205	nucleoporin 205	1.685	1.137	0.825
104458	Rars	arginyl-tRNA synthetase	1.677	1.391	0.949
13135	Dad1	defender against cell death 1	1.67	1.771	0.954
76890	Memo1	mediator of cell motility 1	1.669	1.269	0.999
22146	Tuba1c	tubulin, alpha 1C	1.666	1.352	1.01
27397	Mrpl17	mitochondrial ribosomal protein L17	1.664	1.169	0.918
67398	Srpr	signal recognition particle receptor ('docking protein')	1.664	1.457	0.918
22145	Tuba4a	tubulin, alpha 4A	1.662	1.547	0.926
11519	Add2	adducin 2 (beta)	1.658	1.19	0.942
234138	Tti2	TELO2 interacting protein 2	1.658	1.634	0.933
219189	Vwa8	von Willebrand factor A domain containing 8	1.656	1.361	0.893
66855	Tcf25	transcription factor 25 (basic helix-loop-helix)	1.642	1.243	1.17
18104	Nqo1	NAD(P)H dehydrogenase, quinone 1	1.642	1.567	0.859
238799	Tnpo1	transportin 1	1.639	1.448	0.94
21379	Tbrg4	transforming growth factor beta regulated gene 4	1.637	1.315	1.744
11933	Atp1b3	ATPase, Na+/K+ transporting, beta 3 polypeptide	1.633	1.257	0.906

26897	Acot1	acyl-CoA thioesterase 1	1.632	1.318	0.86
28064	Yipf3	Yip1 domain family, member 3	1.63	1.52	0.789
68581	Tmed10	transmembrane emp24-like trafficking protein 10 (yeast)	1.627	1.397	0.981
272396	Tarsl2	threonyl-tRNA synthetase-like 2	1.626	1.195	0.591
20779	Src	Rous sarcoma oncogene	1.626	1.437	0.923
12874	Cpd	carboxypeptidase D	1.625	1.1	0.759
241274	Pnpla7	patatin-like phospholipase domain containing 7	1.624	1.262	1.136
231380	Uba6	ubiquitin-like modifier activating enzyme 6	1.622	1.237	0.843
224705	Vps52	vacuolar protein sorting 52 (yeast)	1.62	1.541	0.842
52357	Wwc2	WW, C2 and coiled-coil domain containing 2	1.62	1.513	1.077
14866	Gstm5	glutathione S-transferase, mu 5	1.618	1.384	0.763
433256	Acsl5	acyl-CoA synthetase long-chain family member 5	1.617	1.357	0.899
433702	Ncbp1	nuclear cap binding protein subunit 1	1.615	1.315	1.169
70394	Kptn	kaptin	1.613	1.584	1.379
110596	Arhgef28	Rho guanine nucleotide exchange factor (GEF) 28	1.609	2.052	1.25
22247	Umps	uridine monophosphate synthetase	1.606	1.285	0.885
12334	Capn2	calpain 2	1.606	1.459	1.102
98314	D2hgdh	D-2-hydroxyglutarate dehydrogenase	1.604	1.316	1.091
72416	Lrpprc	leucine-rich PPR-motif containing	1.603	1.383	0.865
71449	Mettl13	methyltransferase like 13	1.603	1.291	0.901
22154	Tubb5	tubulin, beta 5 class I	1.6	1.409	1.111
56354	Dnajc7	DnaJ (Hsp40) homolog, subfamily C, member 7	1.597	1.361	1.33
29812	Ndrg3	N-myc downstream regulated gene 3	1.596	1.36	1.215
54128	Pmm2	phosphomannomutase 2	1.595	1.199	0.874
84505	Setdb1	SET domain, bifurcated 1	1.594	1.44	0.682
217342	Ube2o	ubiquitin-conjugating enzyme E2O	1.594	1.275	0.826
99237	Tm9sf4	transmembrane 9 superfamily protein member 4	1.592	1.746	0.849
22273	Uqcrc1	ubiquinol-cytochrome c reductase core protein 1	1.59	1.328	0.713
19087	Prkar2a	protein kinase, cAMP dependent regulatory, type II alpha	1.589	1.473	0.991
269437	Plch1	phospholipase C, eta 1	1.588	1.643	1.05
227446	2310035C23Rik	RIKEN cDNA 2310035C23 gene	1.586	1.261	1.41
52036	Ppp6r3	protein phosphatase 6, regulatory subunit 3	1.584	1.446	1.119
54161	Copg1	coatomer protein complex, subunit gamma 1	1.583	1.353	0.906
67089	Psmc6	proteasome (prosome, macropain) 26S subunit, ATPase, 6	1.583	1.439	0.938
217664	Mgat2	mannoside acetylglucosaminyltransferase 2	1.582	1.204	0.68
237107	Gnl3l	guanine nucleotide binding protein-like 3 (nucleolar)-like	1.582	1.099	0.985
54638	Ccdc22	coiled-coil domain containing 22	1.582	1.393	1.155

268390	Ahsa2	AHA1, activator of heat shock protein ATPase 2	1.581	1.526	0.978
21745	Tep1	telomerase associated protein 1	1.576	1.529	0.801
94230	Cpsf1	cleavage and polyadenylation specific factor 1	1.575	1.316	1.217
330192	Vps37b	vacuolar protein sorting 37B (yeast)	1.575	1.509	0.962
16430	Stt3a	STT3, subunit of the oligosaccharyltransferase complex, homolog A (S. cerevisiae)	1.574	1.458	0.791
67669	l7Rn6	lethal, Chr 7, Rinchik 6	1.573	1.216	0.64
14660	Gls	glutaminase	1.567	1.272	1.027
69590	Gpx8	glutathione peroxidase 8 (putative)	1.566	1.344	0.939
100041230	Hist1h4m	histone cluster 1, H4m	1.564	1.088	1.106
67006	Cisd2	CDGSH iron sulfur domain 2	1.562	1.279	1.157
71766	Raver1	ribonucleoprotein, PTB-binding 1	1.561	1.274	0.841
207352	Sec23ip	Sec23 interacting protein	1.56	1.19	0.976
66191	Ier3ip1	immediate early response 3 interacting protein 1	1.56	1.432	1.031
74334	Ranbp10	RAN binding protein 10	1.555	1.463	0.844
15519	Hsp90aa1	heat shock protein 90, alpha (cytosolic), class A member 1	1.555	1.359	0.897
66335	Atp6v1c1	ATPase, H+ transporting, lysosomal V1 subunit C1	1.555	1.303	1.091
20430	Cyfip1	cytoplasmic FMR1 interacting protein 1	1.552	1.465	0.92
13200	Ddost	dolichyl-di-phosphooligosaccharide-protein glycotransferase	1.551	1.348	0.908
228785	Mylk2	myosin, light polypeptide kinase 2, skeletal muscle	1.551	1.522	1
56480	Tbk1	TANK-binding kinase 1	1.55	1.352	0.945
72584	Cul4b	cullin 4B	1.55	1.357	1.01
12704	Cit	citron	1.548	1.296	1.01
11855	Arhgap5	Rho GTPase activating protein 5	1.547	1.373	0.984
14871	Gstt1	glutathione S-transferase, theta 1	1.547	1.55	0.938
26891	Cops4	COP9 (constitutive photomorphogenic) homolog, subunit 4 (Arabidopsis thaliana)	1.546	1.398	0.909
56347	Eif3c	eukaryotic translation initiation factor 3, subunit C	1.542	1.113	0.905
445007	Nup85	nucleoporin 85	1.542	1.255	1.247
108012	Ap1s2	adaptor-related protein complex 1, sigma 2 subunit	1.541	1.376	1.04
68295	Aar2	AAR2 splicing factor homolog (S. cerevisiae)	1.54	1.178	0.85
65246	Xpo7	exportin 7	1.537	1.345	1.012
72046	Urgcp	upregulator of cell proliferation	1.534	1.225	0.81
13207	Ddx5	DEAD (Asp-Glu-Ala-Asp) box polypeptide 5	1.532	1.247	0.95
18786	Plaa	phospholipase A2, activating protein	1.529	1.227	0.892
16180	Il1rap	interleukin 1 receptor accessory protein	1.528	1.193	1.212

52033	Pbk	PDZ binding kinase	1.526	1.131	0.891
215114	Hip1	huntingtin interacting protein 1	1.526	1.31	0.948
104112	Acly	ATP citrate lyase	1.525	1.211	0.911
98238	Lrrc59	leucine rich repeat containing 59	1.522	1.311	0.839
66882	Bzw1	basic leucine zipper and W2 domains 1	1.522	1.33	0.905
19181	Psmc2	proteasome (prosome, macropain) 26S subunit, ATPase 2	1.522	1.385	0.969
56294	Ptpn9	protein tyrosine phosphatase, non-receptor type 9	1.521	1.412	1.423
15516	Hsp90ab1	heat shock protein 90 alpha (cytosolic), class B member 1	1.519	1.329	0.898
14732	Gpam	glycerol-3-phosphate acyltransferase, mitochondrial	1.519	1.772	1.852
621832	Gm10349	predicted gene 10349	1.518	1.235	0.693
54392	Ncapg	non-SMC condensin I complex, subunit G	1.518	1.312	0.858
11793	Atg5	autophagy related 5	1.517	1.332	0.895
68087	Dcakd	dephospho-CoA kinase domain containing	1.515	1.254	1.009
227693	Zer1	zyg-11 related, cell cycle regulator	1.515	1.524	1.217
54160	Copg2	coatomer protein complex, subunit gamma 2	1.514	1.457	0.986
14156	Fen1	flap structure specific endonuclease 1	1.514	1.286	1.281
59053	Fam203a	family with sequence similarity 203, member A	1.514	1.525	0.988
270076	Gcdh	glutaryl-Coenzyme A dehydrogenase	1.514	1.539	1.094
67618	Aasdhppt	aminoadipate-semialdehyde dehydrogenase-phosphopantetheinyl transferase	1.512	1.162	1.129
74868	Tmem65	transmembrane protein 65	1.512	1.596	1.189
16881	Lig1	ligase I, DNA, ATP-dependent	1.507	1.045	0.838
56401	Lepre1	leprecan 1	1.507	1.384	0.963
74122	Tmem43	transmembrane protein 43	1.506	1.375	0.9
58184	Rqcd1	rcd1 (required for cell differentiation) homolog 1 (S. pombe)	1.505	1.482	0.96
75646	Rai14	retinoic acid induced 14	1.505	1.66	1.096
66971	Cdk5rap1	CDK5 regulatory subunit associated protein 1	1.505	1.512	1.188
13367	Diap1	diaphanous homolog 1 (Drosophila)	1.504	1.45	0.91
13972	Gnb1l	guanine nucleotide binding protein (G protein), beta polypeptide 1-like	1.504	1.329	0.982
68015	Trap1	TNF receptor-associated protein 1	1.5	1.288	0.916
