# Supplementary material for: Proteomic Analysis Implicates Dominant Alterations of RNA Metabolism and the Proteasome Pathway in the Cellular Response to Carbon-Ion Irradiation
Source: PLoS One. 2016 Oct 6;11(10):e0163896. doi: 10.1371/journal.pone.0163896 (PMC5053480; doi:10.1371/journal.pone.0163896)
Supplement: S2 Table — (PDF) [file pone.0163896.s003.pdf]

S2 Table The upregulated proteins by 2 Gy of 12.6 KeV/μm carbon ions and their overlaps with other irradiation groups.

The upregulated proteins by 2 Gy irradiation of plateau carbon ion with the LET of 12.6 KeV/μm, and their partial overlaps to other irradiation groups (LET12.6-0.2 Gy and LET31.5-2Gy) (black characters on pink background)

Mouse Gene ID	Symbol	Description	Changed fold		
			LET12.6		LET31.5
			2Gy	0.2Gy	2Gy
272538	Tango6	transport and golgi organization 6	3.173	2.25	1.818
69150	Snx4	sorting nexin 4	2.475	2.299	0.942
75786	Ckap5	cytoskeleton associated protein 5	2.218	2.326	1.332
217337	Srp68	signal recognition particle 68	2.124	2.295	1.081
110596	Arhgef28	Rho guanine nucleotide exchange factor (GEF) 28	2.052	1.609	1.25
20669	Sox14	SRY-box containing gene 14	2.014	0.336	2.988
69155	1810030O07Rik	RIKEN cDNA 1810030O07 gene	1.925	2.567	1.243
216363	Rab3ip	RAB3A interacting protein	1.894	2.855	1.043
217995	Heatr1	HEAT repeat containing 1	1.89	1.694	1.07
69543	Capns2	calpain, small subunit 2	1.878	1.476	1.145
66092	Ghitm	growth hormone inducible transmembrane protein	1.823	1.701	1.132
67542	Cog6	component of oligomeric golgi complex 6	1.81	2.37	1.098
433956	Heatr2	HEAT repeat containing 2	1.807	1.691	1.187
259279	Tubgcp3	tubulin, gamma complex associated protein 3	1.796	1.442	0.789
14732	Gpam	glycerol-3-phosphate acyltransferase, mitochondrial	1.772	1.519	1.852
13135	Dad1	defender against cell death 1	1.771	1.67	0.954
99237	Tm9sf4	transmembrane 9 superfamily protein member 4	1.746	1.592	0.849
234663	Dync1li2	dynein, cytoplasmic 1 light intermediate chain 2	1.726	1.453	1.825
70549	Tln2	talin 2	1.72	1.826	1.003
109168	Atl3	atlastin GTPase 3	1.706	1.954	0.684
227737	Fam129b	family with sequence similarity 129, member B	1.695	1.77	0.676
20589	Ighmbp2	immunoglobulin mu binding protein 2	1.677	1.479	1.176
394434	Ugt1a9	UDP glucuronosyltransferase 1 family, polypeptide A9	1.671	1.453	3.74
75646	Rai14	retinoic acid induced 14	1.66	1.505	1.096
269437	Plch1	phospholipase C, eta 1	1.643	1.588	1.05
226043	Cbwd1	COBW domain containing 1	1.643	1.866	1.62
117586	A1bg	alpha-1-B glycoprotein	1.637	0.896	4.095
234138	Tti2	TELO2 interacting protein 2	1.634	1.658	0.933
234733	Ddx19b	DEAD (Asp-Glu-Ala-Asp) box polypeptide 19b	1.633	1.872	0.798

55935	Fnbp4	formin binding protein 4	1.628	1.22	1.004
56041	Uso1	USO1 vesicle docking factor	1.627	1.735	1.05
110279	Bcr	breakpoint cluster region	1.617	1.202	1.523
69276	Sec62	SEC62 homolog (S. cerevisiae)	1.596	1.816	0.904
74868	Tmem65	transmembrane protein 65	1.596	1.512	1.189
70394	Kptn	kaptin	1.584	1.613	1.379
14431	Gamt	guanidinoacetate methyltransferase	1.582	1.285	0.915
225326	Pik3c3	phosphoinositide-3-kinase, class 3	1.581	0.698	2.583
28035	Usp39	ubiquitin specific peptidase 39	1.574	0.928	1.076
18104	Nqo1	NAD(P)H dehydrogenase, quinone 1	1.567	1.642	0.859
72508	Rps6kb1	ribosomal protein S6 kinase, polypeptide 1	1.566	1.439	1.091
18753	Prkcd	protein kinase C, delta	1.565	1.739	1.201
11773	Ap2m1	adaptor-related protein complex 2, mu 1 subunit	1.56	1.34	1.188
29816	Hip1r	huntingtin interacting protein 1 related	1.558	1.371	1.113
14871	Gstt1	glutathione S-transferase, theta 1	1.55	1.547	0.938
67673	Tceb2	transcription elongation factor B (SIII), polypeptide 2	1.548	1.746	1.39
22145	Tuba4a	tubulin, alpha 4A	1.547	1.662	0.926
56207	Uchl5	ubiquitin carboxyl-terminal esterase L5	1.543	1.884	0.984
224705	Vps52	vacuolar protein sorting 52 (yeast)	1.541	1.62	0.842
270076	Gcdh	glutaryl-Coenzyme A dehydrogenase	1.539	1.514	1.094
67345	Herc4	hect domain and RLD 4	1.53	1.471	0.825
21745	Tep1	telomerase associated protein 1	1.529	1.576	0.801
67955	Sugt1	SGT1, suppressor of G2 allele of SKP1 (S. cerevisiae)	1.526	1.449	1.075
268390	Ahsa2	AHA1, activator of heat shock protein ATPase 2	1.526	1.581	0.978
59053	Fam203a	family with sequence similarity 203, member A	1.525	1.514	0.988
227693	Zer1	zyg-11 related, cell cycle regulator	1.524	1.515	1.217
228785	Mylk2	myosin, light polypeptide kinase 2, skeletal muscle	1.522	1.551	1
28064	Yipf3	Yip1 domain family, member 3	1.52	1.63	0.789
57342	Parva	parvin, alpha	1.519	1.713	1.145
80748	BC004004	cDNA sequence BC004004	1.518	1.888	1.293
75292	Prkd3	protein kinase D3	1.513	1.43	1.294
52357	Wwc2	WW, C2 and coiled-coil domain containing 2	1.513	1.62	1.077
66971	Cdk5rap1	CDK5 regulatory subunit associated protein 1	1.512	1.505	1.188
75475	Oplah	5-oxoprolinase (ATP-hydrolysing)	1.51	0.82	0.847
14679	Gnai3	guanine nucleotide binding protein (G protein), alpha inhibiting 3	1.509	1.421	1.312
330192	Vps37b	vacuolar protein sorting 37B (yeast)	1.509	1.575	0.962
224805	Aars2	alanyl-tRNA synthetase 2, mitochondrial (putative)	1.504	1.409	0.925
211446	Exoc3	exocyst complex component 3	1.502	1.273	0.919

20166	Rtkn	rhotekin	1.502	1.71	0.939
14712	Gnpat	glyceronephosphate O-acyltransferase	1.5	1.191	1.051
67130	Ndufa6	NADH dehydrogenase (ubiquinone) 1 alpha subcomplex, 6 (B14)	1.5	1.693	1.25
