# Supplementary material for: Proteomic Analysis Implicates Dominant Alterations of RNA Metabolism and the Proteasome Pathway in the Cellular Response to Carbon-Ion Irradiation
Source: PLoS One. 2016 Oct 6;11(10):e0163896. doi: 10.1371/journal.pone.0163896 (PMC5053480; doi:10.1371/journal.pone.0163896)
Supplement: S3 Table — (PDF) [file pone.0163896.s004.pdf]

S3 Table The upregulated proteins by 2 Gy of 31.5 KeV/μm carbon ion beams and their overlaps with other irradiation groups

The upregulated proteins by 2 Gy irradiation of carbon ion beams with the LET of 31.5 KeV/μm, and their partial overlaps with other irradiation groups (LET12.6- 0.2 Gy and 2Gy) (black characters on pink background ground)

Mouse Gene ID	Symbol	Description	Changed fold		
			LET31.5	LET12.6	
			2Gy	0.2Gy	2Gy
117586	A1bg	alpha-1-B glycoprotein	4.095	0.896	1.637
394434	Ugt1a9	UDP glucuronosyltransferase 1 family, polypeptide A9	3.74	1.453	1.671
83561	Tdrd1	tudor domain containing 1	3.707	0.497	1.22
117599	Helb	helicase (DNA) B	3.677	1.445	1.288
68597	Ccdc167	coiled-coil domain containing 167	3.217	1.044	1.2
20669	Sox14	SRY-box containing gene 14	2.988	0.336	2.014
232288	Frmd4b	FERM domain containing 4B	2.745	0.369	0.526
225326	Pik3c3	phosphoinositide-3-kinase, class 3	2.583	0.698	1.581
15248	Hic1	hypermethylated in cancer 1	2.544	0.791	1.085
22041	Trf	transferrin	2.515	0.527	0.932
70753	6330415B21Rik	RIKEN cDNA 6330415B21 gene	2.456	0.486	0.634
100045367	LOC100045367	60S ribosomal protein L19-like	2.324	0.833	1.249
100040259	Gm16379	predicted pseudogene 16379	2.204	0.618	1.379
12842	Col1a1	collagen, type I, alpha 1	2.141	0.307	0.498
51796	Srrm1	serine/arginine repetitive matrix 1	2.134	0.626	1.015
14148	Fdx1	ferredoxin 1	2.118	0.483	1.154
57441	Gmnn	geminin	2.105	0.646	0.731
67848	Ddx55	DEAD (Asp-Glu-Ala-Asp) box polypeptide 55	2.07	0.248	0.441
101314	Brk1	BRICK1, SCAR/WAVE actin-nucleating complex subunit	2.049	1.184	1.303
15168	Hcn3	hyperpolarization-activated, cyclic nucleotide-gated K+ 3	2.037	0.894	1.193
226162	Dpcd	deleted in primary ciliary dyskinesia	2.025	1.416	1.492
22130	Ttf1	transcription termination factor, RNA polymerase I	1.967	0.802	0.941
56350	Arl3	ADP-ribosylation factor-like 3	1.923	0.44	0.63
53861	Zranb2	zinc finger, RAN-binding domain containing 2	1.921	0.463	0.789
109905	Rap1a	RAS-related protein-1a	1.897	0.688	0.826
245828	Trappc1	trafficking protein particle complex 1	1.889	1.159	1.44
67980	Gnpda2	glucosamine-6-phosphate deaminase 2	1.87	0.51	0.627
15354	Hmgb3	high mobility group box 3	1.864	0.812	1.244
14732	Gpam	glycerol-3-phosphate acyltransferase, mitochondrial	1.852	1.519	1.772
74104	Abcb6	ATP-binding cassette, sub-family B	1.847	1.068	1.119

		(MDR/TAP), member 6			
234582	Ccdc102a	coiled-coil domain containing 102A	1.829	0.383	0.573
57748	Jmy	junction-mediating and regulatory protein	1.827	1.001	1.251
234663	Dync1li2	dynein, cytoplasmic 1 light intermediate chain 2	1.825	1.453	1.726
272538	Tango6	transport and golgi organization 6	1.818	2.25	3.173
66510	Rnf181	ring finger protein 181	1.797	0.74	1.015
22393	Wfs1	Wolfram syndrome 1 homolog (human)	1.796	1.209	1.403
73046	Glrx5	glutaredoxin 5 homolog (S. cerevisiae)	1.787	0.641	0.83
78655	Eif3j1	eukaryotic translation initiation factor 3, subunit J1	1.772	0.487	0.678
18611	Pea15a	phosphoprotein enriched in astrocytes 15A	1.764	0.737	1.167
12394	Runx1	runt related transcription factor 1	1.752	0.591	0.861
381293	Kif14	kinesin family member 14	1.752	1.141	1.326
18634	Pex7	peroxisomal biogenesis factor 7	1.746	0.601	0.654
21379	Tbrg4	transforming growth factor beta regulated gene 4	1.744	1.637	1.315
67938	Myl12b	myosin, light chain 12B, regulatory	1.741	0.778	0.804
68045	2700060E02Rik	RIKEN cDNA 2700060E02 gene	1.738	0.639	0.914
66801	Prkrip1	Prkr interacting protein 1 (IL11 inducible)	1.732	0.557	0.952
624056	Ctsm-ps2	cathepsin M, pseudogene 2	1.725	0.607	0.806
50876	Tmod2	tropomodulin 2	1.724	1.086	1.084
66094	Lsm7	LSM7 homolog, U6 small nuclear RNA associated (S. cerevisiae)	1.713	0.511	0.719
58231	Stk4	serine/threonine kinase 4	1.711	0.855	0.931
219024	Tmem55b	transmembrane protein 55b	1.706	1.23	1.183
100048759	LOC100048759	complement C3-like	1.701	0.619	0.805
66419	Mrpl11	mitochondrial ribosomal protein L11	1.7	1.106	0.963
110391	Qdpr	quinoid dihydropteridine reductase	1.699	0.597	0.674
226604	Gm4847	predicted gene 4847	1.697	1.128	1.25
106572	Rab31	RAB31, member RAS oncogene family	1.693	1.079	1.039
26951	Zw10	zw10 kinetochore protein	1.689	1.32	1.273
68636	Fahd1	fumarylacetoacetate hydrolase domain containing 1	1.688	0.622	0.942
56372	1110004F10Rik	RIKEN cDNA 1110004F10 gene	1.683	0.513	0.784
83703	Dbr1	debranching enzyme homolog 1 (S. cerevisiae)	1.677	0.656	0.789
74498	Gorasp1	golgi reassembly stacking protein 1	1.677	1.117	1.303
72065	Rap2c	RAP2C, member of RAS oncogene family	1.671	0.871	1.009
12540	Cdc42	cell division cycle 42	1.67	0.615	0.819
19353	Rac1	RAS-related C3 botulinum substrate 1	1.662	0.83	0.98
22004	Tpm2	tropomyosin 2, beta	1.659	0.575	0.854
12845	Comp	cartilage oligomeric matrix protein	1.654	0.034	0.076
29877	Hdgfrp3	hepatoma-derived growth factor, related protein 3	1.654	0.474	0.629
230935	Dnajc11	DnaJ (Hsp40) homolog, subfamily C, member 11	1.649	1.038	1.077

12321	Calu	calumenin	1.633	0.426	0.774
17836	Mug1	murinoglobulin 1	1.632	0.52	1.011
68097	Dynll2	dynein light chain LC8-type 2	1.629	0.526	0.712
545192	Baiap3	BAI1-associated protein 3	1.629	0.374	0.827
193740	Hspa1a	heat shock protein 1A	1.628	0.489	0.562
18476	Pafah1b3	platelet-activating factor acetylhydrolase, isoform 1b, subunit 3	1.625	0.797	0.933
170760	Acbd3	acyl-Coenzyme A binding domain containing 3	1.624	0.865	0.959
73112	Abracl	ABRA C-terminal like	1.624	1.119	1.036
19038	Ppic	peptidylprolyl isomerase C	1.623	0.652	0.699
22608	Ybx1	Y box protein 1	1.62	0.537	0.717
226043	Cbwd1	COBW domain containing 1	1.62	1.866	1.643
22070	Tpt1	tumor protein, translationally-controlled 1	1.619	0.682	0.769
66988	Lap3	leucine aminopeptidase 3	1.616	0.59	0.85
192160	Casc3	cancer susceptibility candidate 3	1.616	0.847	1.213
109658	Txlna	taxilin alpha	1.61	0.554	0.858
101471	Phrf1	PHD and ring finger domains 1	1.61	0.987	1.138
56205	Ensa	endosulfine alpha	1.605	0.545	1.012
74764	Klc4	kinesin light chain 4	1.599	1.057	1.055
215351	Senp6	SUMO/sentrin specific peptidase 6	1.597	0.908	0.989
15364	Hmga2	high mobility group AT-hook 2	1.59	0.831	0.845
20198	S100a4	S100 calcium binding protein A4	1.588	0.586	0.988
66902	Mtap	methylthioadenosine phosphorylase	1.587	0.49	0.759
14227	Fkbp2	FK506 binding protein 2	1.582	0.508	0.874
15361	Hmga1	high mobility group AT-hook 1	1.582	0.736	0.973
77697	Mmab	methylmalonic aciduria (cobalamin deficiency) type B homolog (human)	1.581	0.827	1.031
66070	Cwc15	CWC15 homolog (S. cerevisiae)	1.581	0.827	1.065
14924	Magi1	membrane associated guanylate kinase, WW and PDZ domain containing 1	1.577	0.334	0.42
12261	C1qbp	complement component 1, q subcomponent binding protein	1.574	0.3	0.354
381045	Ccdc58	coiled-coil domain containing 58	1.574	0.5	0.86
20851	Stat5b	signal transducer and activator of transcription 5B	1.573	1.096	1.21
19248	Ptpn12	protein tyrosine phosphatase, non-receptor type 12	1.57	1.116	1.138
69694	Tatdn1	TatD DNase domain containing 1	1.567	0.588	0.564
227099	Pms1	postmeiotic segregation increased 1 (S. cerevisiae)	1.565	1.201	1.221
66138	Wbscr22	Williams Beuren syndrome chromosome region 22	1.564	0.58	0.599
19072	Prep	prolyl endopeptidase	1.563	1.019	1.036
100505031	LOC100505031	40S ribosomal protein SA-like	1.559	0.547	0.593
192174	Rwdd4a	RWD domain containing 4A	1.557	0.615	0.747
382769	Gm5196	chromobox homolog 3 pseudogene	1.555	0.511	0.578

17448	Mdh2	malate dehydrogenase 2, NAD (mitochondrial)	1.555	0.486	0.634
22210	Ube2b	ubiquitin-conjugating enzyme E2B	1.553	0.586	0.71
67886	Camsap2	calmodulin regulated spectrin-associated protein family, member 2	1.548	0.997	1.133
240614	Ranbp6	RAN binding protein 6	1.546	0.577	0.885
17975	Ncl	nucleolin	1.545	0.574	0.697
234023	Arglu1	arginine and glutamate rich 1	1.544	0.47	0.733
98660	Atp1a2	ATPase, Na+/K+ transporting, alpha 2 polypeptide	1.539	0.34	0.384
56309	Mycbp	c-myc binding protein	1.537	0.626	0.913
19231	Ptma	prothymosin alpha	1.536	0.536	0.793
109075	Exosc4	exosome component 4	1.534	0.621	0.623
237806	Dnahc9	dynein, axonemal, heavy chain 9	1.534	0.412	0.846
232989	Hnrnpul1	heterogeneous nuclear ribonucleoprotein U-like 1	1.531	0.647	0.766
623286	Gm6415	predicted pseudogene 6415	1.531	0.477	0.807
68219	Nudt21	nudix (nucleoside diphosphate linked moiety X)-type motif 21	1.529	0.626	0.718
66282	Tma16	translation machinery associated 16 homolog (S. cerevisiae)	1.529	0.695	0.824
19326	Rab11b	RAB11B, member RAS oncogene family	1.527	0.594	0.9
19684	Rdx	radixin	1.525	0.472	0.586
66514	Asrgl1	asparaginase like 1	1.525	0.433	0.786
14297	Fxn	frataxin	1.524	0.635	0.699
66983	Zfp830	zinc finger protein 830	1.524	0.595	0.886
110279	Bcr	breakpoint cluster region	1.523	1.202	1.617
665563	Mthfd2l	methylenetetrahydrofolate dehydrogenase (NADP+ dependent) 2-like	1.521	0.724	0.984
11287	Pzp	pregnancy zone protein	1.514	0.654	0.601
12412	Cbx1	chromobox 1	1.513	0.562	0.655
223527	Eny2	enhancer of yellow 2 homolog (Drosophila)	1.512	0.561	0.755
11980	Atp8a1	ATPase, aminophospholipid transporter (APLT), class I, type 8A, member 1	1.511	1.147	1.1
321022	Cdv3	carnitine deficiency-associated gene expressed in ventricle 3	1.51	0.454	0.758
11641	Akap2	A kinase (PRKA) anchor protein 2	1.507	0.887	1.119
100041106	Gm3141	predicted gene 3141	1.502	1.03	0.805
223690	Ankrd54	ankyrin repeat domain 54	1.5	0.876	0.968
